# Determination of Lubrication Layer Thickness and Its Effect on Concrete Pumping Pressure

**DOI:** 10.3390/ma17205136

**Published:** 2024-10-21

**Authors:** Rong Deng, Tong Ye, Zhiwei Ye

**Affiliations:** School of Mechanical Engineering, University of South China, Hengyang 421100, China; yetong0805@163.com (T.Y.); yezhiwei0601@163.com (Z.Y.)

**Keywords:** fresh concrete, pressure per unit length, horizontal pipe, simulation

## Abstract

The flow of six kinds of fresh concrete under different flow rates and lubrication layer thickness (T_LL_) values in the horizontal pipe was numerically simulated. The influence of the T_LL_ on the pressure per unit length (P_L_) was analyzed. It was determined that the formation of the lubrication layer (LL) significantly reduces the P_L_ in concrete pumping. As the T_LL_ increased, the P_L_ decreased. However, the degree of reduction in the P_L_ gradually decreased as the T_LL_ increased. Relating the simulated P_L_ with the experimental P_L_, the size of the T_LL_ was obtained, which was between 1 and 3 mm. The minimum and maximum were 1.23 and 2.58 mm, respectively, and the average value was 1.97 mm. The strength (S24, S50), the size of the aggregate (A10, A20, A25), and the flow rate of pumping all affected the T_LL_. The type of fresh concrete and the flow rate of pumping significantly affected the P_L_, which impacted the T_LL_. However, the T_LL_ also impacted the P_L_. Finally, this made the T_LL_ change within a certain range. When P_L_ > 14,000 Pa/m, 2 mm < T_LL_< 3 mm; on the other hand, 1 mm < T_LL_< 2 mm. Therefore, we can use CFD to simulate the flow of all types of concrete in the actual pumping pipeline with a T_LL_ of 2 mm to obtain their pumping pressure and guide the actual construction.

## 1. Introduction

Concrete, as the most widely used engineering material, is extensively used in the construction of urban infrastructure, roads, bridges, and nuclear reactors. Pumping technology is a construction method used to complete the crucial tasks of concrete transportation and pouring, offering advantages such as speed, timeliness, quality assurance, and reduced labor consumption [1,2]. Especially for some large-scale reinforced concrete structures that use substantial amounts of concrete, high-rise buildings, narrow sites, and construction sites with obstacles, the concrete pumping technology is particularly effective [3,4,5]. Despite extensive experience with concrete pumping, several problems still occur during the actual construction process, including concrete segregation, pipeline blockage, and wear [6]. These problems greatly increase the pumping pressure and can even cause the pumping pipeline to rupture, disrupting the orderly progress of construction and compromising the strength and durability of hardened concrete. Concrete is a multiphase and multi-scale composite material. Its mechanical properties change with time, temperature, humidity, and stress state, showing the evolution between the elastic, viscous, and plastic phases [7,8]. Rheology is the study of the dynamics in the evolution of the viscoelastic–plastic behavior of concrete. It helps identify the changes in concrete during the fresh mixing stage by analyzing the interactions between the different phases in the slurry. Employing the rheology theory to study the rheological behavior of concrete, the pumping construction can be better guided. Thus, studying the rheological properties of fresh concrete within the pump pipe is crucial. This research holds significant value in predicting the pressure requirements for concrete pumping. In the process of pumping, pressure is the key parameter that determines the efficiency of pumping. The main factors affecting the pressure during concrete pumping include rheological parameters, pumping flow rate, etc. Therefore, predicting the pumping pressure is crucial to ensuring a smooth and effective pumping process.

A thin layer of several millimeters may be formed on the pipe wall in the process of pumping; this is the lubricating layer (LL) [4,9,10]. The formation, size, and performance of the LL are strongly correlated with the pumping performance of fresh concrete and significantly impact the pumping pressure. It was shown that the rheological properties and thickness of the LL can effectively predict the pumping performance of concrete. The friction between the LL and the pipe wall, which is directly related to the composition of the LL, plays a crucial role in the pumpability of fresh concrete. As a result, the LL plays a key role in the flow process of concrete within the pipe, and with the increase in the T_LL_, the pipeline pressure gradually decreases [11,12,13,14,15,16,17]. At present, there are two main methods to characterize the LL. Through the use of tribology, the properties of the LL were described by Kaplan et al. [18]. The LL was described by other scholars as the relative slip between the concrete near the pipe wall and the pipe wall. The slip velocity was introduced to deal with the influence of the LL on the pumping [19,20,21]. It was agreed that shear-induced particle migration is the cause of the formation of the LL in the pipe wall during pumping [11,19,22]. The rheological properties of the LL were measured mainly using sliding-tube rheometers [20] and tribometers [21]. The sliding-tube rheometer was used to evaluate the performance of the LL during concrete pumping and to study its influence on the pumping performance of concrete. Numerous studies have shown that the composition and rheological properties of the LL are similar to those of the mortar in fresh concrete [10,23,24]. Le et al. [24] equated the measured rheological parameters of the mortar in fresh concrete to those of the LL to study the influence of the LL on concrete flow in the pump pipe through numerical simulation. It was shown that there is a good correlation between the experiment and the numerical simulation. Accordingly, the rheological properties of the LL could approximately be represented by measuring the rheological properties of mortar in fresh concrete. At present, ultrasonic velocity profiling (UVP) [23,25] and particle image velocimetry (PIV) [24] are mainly used to measure the T_LL_ of fresh concrete during pumping. Studies showed that the T_LL_ ranged from 2 mm to 8 mm and was influenced by the mix ratio of fresh concrete and the inner diameter of the pump pipe [26,27,28,29]. It was also believed that the T_LL_, ranging from 1 mm to 9 mm, is related to the volume of cement slurry, the water–cement ratio, and the content of superplasticizer [13].

The pumping pressure of fresh concrete is influenced by the LL. Thus, numerous scholars have proposed and established models for predicting the pumping pressure. Kaplan’s model [18] was more classic and closer to the actual situation among the various models considering the influence of the LL on the pumping performance of fresh concrete [30]. By comparing the shear stress of fresh concrete near the pipe wall to its yield stress, two models were established to predict the relationship between pumping pressure and flow. Meanwhile, the Kaplan model can also describe the influence of the properties of the LL on the pumping pressure of fresh concrete. Choi et al. [12] found that the rheological parameters of the LL were smaller than those of concrete and that its rheological properties significantly affected the pumping pressure. The formation of the LL was crucial to the pumping pressure. Without the formation of the LL on the pipe wall during the pumping process, the pumping pressure would greatly increase [31,32]. Pumping pressure would be significantly decreased with the increase in T_LL_ [33]. Feys et al. [34] pointed out that the pressure of fresh concrete during pumping could be precisely evaluated by measuring the rheological properties and T_LL_ combined with the rheological characteristics of fresh concrete. Choi et al. [10] simulated the pumping process of fresh concrete using CFD, considering the properties of the LL. The results showed that the numerical simulation could accurately predict the P_L_ of fresh concrete during pumping. Chen et al. [17] also simulated the flow process of fresh concrete in the pipe using CFD and precisely estimated the pumping pressure required to form various T_LL_ conditions on the pipe wall.

In conclusion, the pumping performance is significantly affected by the LL formed on the pipe wall during the pumping of fresh concrete. However, due to the lack of appropriate measurement methods, it is difficult to directly and accurately measure the T_LL_. Therefore, to precisely estimate the pumping P_L_ to guide the actual construction more effectively, the numerical simulation of fresh concrete pumping, as well as considering the influence of the T_LL_ and obtaining its size, is crucial. In this paper, the flow of six kinds of fresh concrete under different flow rates and T_LL_ conditions in the horizontal pipe was simulated. Firstly, the feasibility of CFD to simulate the rheology of fresh concrete using the Bingham rheological model was verified by the experiments and numerical simulations of the slump test, L-box flow test, and V-funnel test. Then, the influence of the T_LL_ on the P_L_ was simulated. Combining the simulation P_L_ and the experimental P_L_, the size of the T_LL_ was obtained. The relationship between the actual size of the T_LL_ and the P_L_ was discussed. Finally, some important conclusions were given.

## 2. Materials and Methods

### 2.1. Characteristics of Initial Materials

In this paper, the flow properties of fresh C30 concrete were tested and calibrated. The concrete was supplied by a commercial concrete company. Its composition and proportions are shown in Table 1. C30 means the compressive strength of concrete is 30 MPa after 28 days of curing. The flow behavior of fresh C30 concrete was assumed to be non-Newtonian following the Bingham law [35], which characterizes the yield stress and plastic viscosity [35,36]. The rheological equation is shown as follows:

(1) $$\tau =\tau_{0}+\eta \gamma$$

**Figure 1:**
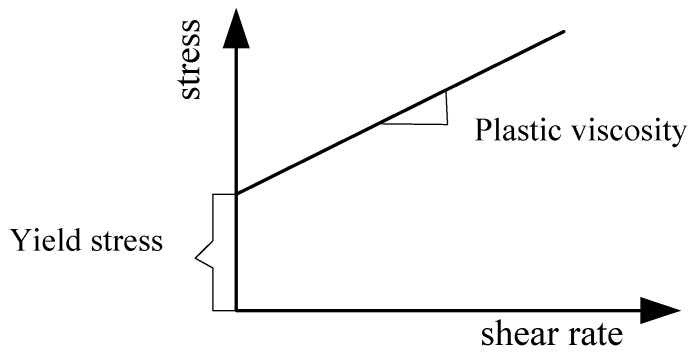
Bingham fluid.

τ is the shear stress, $\tau_{0}$ is the yield stress, η is the viscosity, and γ is the shear rate. The relationship curve between shear stress and shear rate is shown in Figure 1, where the influence of rheological parameters (yield stress and plastic viscosity) on the flow of fresh concrete is described. Fresh concrete remains stationary when the shear stress is less than its yield stress. It immediately starts to flow once its yield stress is exceeded. Once flowing, the flow velocity of the concrete is influenced by its plastic viscosity.

**Table 1:** Content of each component of fresh C30 concrete.

Concrete Grade	Water–Cement Ratio	Water	Cement	Secondary Fly Ash	Sand	Stone	Water-Reducing Agent
C30	0.42	164	300	90	900	1080	3.2

In the CFD simulation, the fresh concrete was regarded as an incompressible fluid. Throughout the flow process, the concrete was assumed to be isothermal, with the energy equation disregarded. The flow of the concrete was described using the Navier–Stokes equation. In the Cartesian coordinate system, the differential forms of the mass conservation equation and momentum equation were expressed by Equations (2) and (3), respectively.

(2) $$\frac{\partial \rho }{\partial t}+\nabla \cdot \left(\rho \nu \right)=0$$

(3) $$\frac{\partial (\rho \nu )}{\sigma t}+\nabla \cdot \left(\rho \nu \nu \right)=-\nabla p+\nabla \cdot \left(\tau \right)+\rho g+F$$

where *p* is the static pressure on the fluid, ρ is the density, ν is the velocity vector, τ is the stress tensor, and *F* is the generalized source term.

### 2.2. Experiment

Rheological properties mainly include the flow ability, filling ability, passing ability, segregation resistance, etc. This measurement method mainly depends on its relationship to workability. Additionally, factors such as cost, site conditions, and the advantages and disadvantages of each test (e.g., economy, convenience, operability, and actual situation) should also be considered. In this study, the slump, L-box test, and V-funnel tests were used to calibrate the rheological parameters (yield stress and plastic viscosity) of fresh C30 concrete based on the CFD.

**Figure 2:**
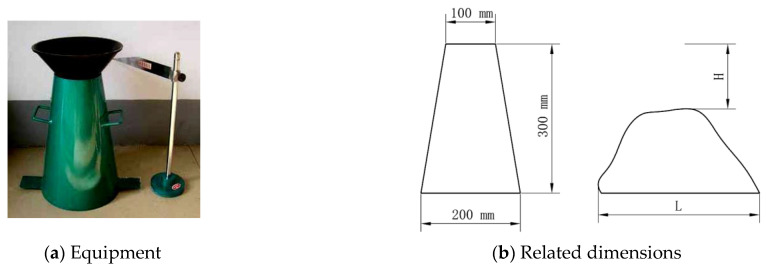
The equipment and related dimensions of the slump test.

The slump test is mainly used to measure the flowability of fresh concrete. Owing to simple equipment and operation, it is practical in laboratories and construction sites. In the slump test, the slump height H (mm) and expansion width L_1_ × L_2_ (mm) are key indicators for assessing the flowability of fresh concrete. The basic device of the slump test is shown in Figure 2a. The dimensions are given in Figure 2b, with top and bottom diameters of 100 and 200 mm, respectively, and a height of 300 mm. H represents the slump height, and L represents the expansion width. The slump test was carried out several times, and the results are summarized in Table 2.

**Figure 3:**
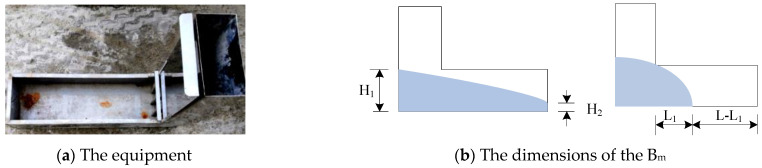
The equipment and test dimensions of the L-box test.

The L-box flow meter is mainly used to evaluate the passing ability of fresh concrete, that is, the ability to cross dense steel bars. It consists of an L-shaped box made from steel plate, featuring a movable door for partitioning and a detachable steel mesh, as depicted in Figure 3a. In the L-box flow test, the flow index (B_m_) is used to quantitatively describe the flow performance of fresh concrete. B_m_ is defined in two ways: when the fresh concrete can flow to the rightmost end of the horizontal box, B_m_ = H_2_/H_1_; otherwise, B_m_ = (L_1_ − L)/L. The parameters L_1_, L_2_, H_1_, and H_2_ are defined in Figure 3b. When −1 ≤ B_m_ ≤ 1, a larger value of B_m_ indicates better flowability of the fresh concrete. Multiple L-box flow tests were conducted, with the results summarized in Table 3.

The V-funnel test is used to assess the viscosity and segregation resistance of fresh concrete and is suitable for all grades of fresh concrete. The evaluation index is the flowing time T_v_ (s), which is defined as the time from the opening of the valve until the fresh concrete has completely exited the V-funnel. The V-funnel test was carried out several times, with the results summarized in Table 4.

### 2.3. Simulation

**Figure 4:**
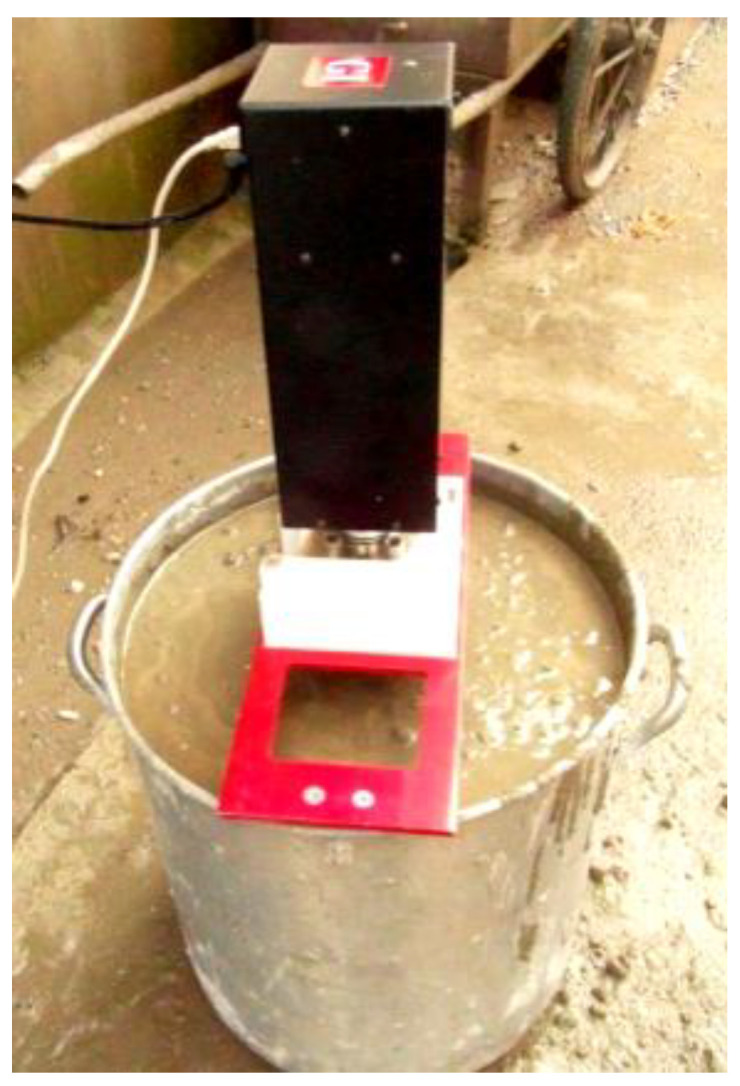
Measuring the rheological parameters of fresh concrete using the ICAR rheometer.

**Figure 5:**
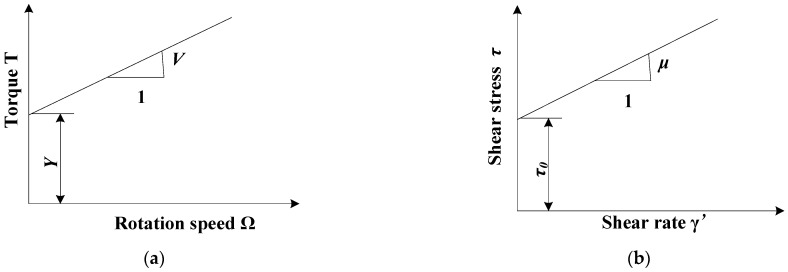
Torque and speed are converted to rheological parameters: (**a**) curve of rotation speed and torque; (**b**) curve of shear stress and shear rate.

The slump, L-box, and V-funnel tests were 3D modeled and meshed, and boundary conditions were set according to the actual situation. A two-phase flow volume of fluid (VOF) model [37] was used to simulate the flow behavior of fresh concrete in these tests. The first and second phases were air and fresh concrete, respectively. The rheological model of fresh C30 concrete was characterized by the Bingham model, with a density of 2400 kg/m^3^. The rheological parameters were measured using the ICAR rheometer, as shown in Figure 4. The experimental procedure is not described in detail here. The rheological parameters were obtained by linearly fitting the torque and rotation speed to obtain the slope and intercept, which were calculated using the Reiner–Riwlin formula. The relationship between rotation speed, torque, yield stress, and plastic viscosity is shown in Figure 5.

**Figure 6:**
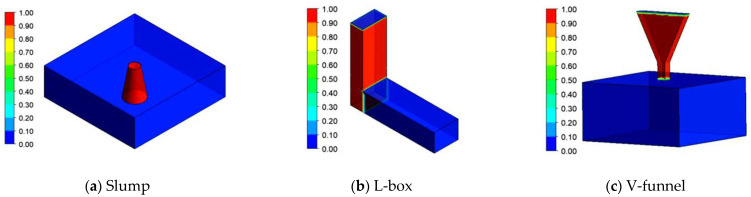
The initial distribution of fresh concrete in the flow tests.

The simulated initial states of fresh concrete in the slump, L-box, and V-funnel tests are shown in Figure 6, where ‘0’ indicates only air, ‘1’ indicates only fresh concrete, and ‘0–1’ represents a mix of both air and fresh concrete at any interface.

During the pumping process, the formation of the LL significantly promotes the pumping process of the fresh concrete. Secrieru et al. [32] stated that concrete cannot be pumped without the formation of the LL at the interface between the concrete and the pipe wall. Kaplan et al. [18] found that the LL has a thickness ranging from approximately 1 to 5 mm. Ngo et al. [13,14] stated that the T_LL_ for different concrete mixtures varies between 1 and 9 mm. It was also reported that the T_LL_ ranges from 2 to 8 mm [26,27,28,29] or from 1 to 9 mm [13].

In this study, to determine the size of the T_LL_ and its effect on the flow of fresh concrete in pipes, various horizontal pipes were modeled with a diameter of 125 mm, a length of 2000 mm, and T_LL_ values of 0, 1, 2, 3, 4, 6, and 8 mm. Then, they were meshed, and the boundary conditions were set according to the actual working conditions. Finally, the flow process of fresh concrete in these pipes was simulated using CFD. The density of the central concrete was set at 2400 kg/m^3^. The properties of the LL are similar to those of the mortar of the pumped concrete [10]; thus, its density was set at 1600 kg/m^3^. The flow of six kinds of concrete with different rheological parameters under certain flow rates in the horizontal pipe was simulated. The rheological parameters of the center concrete and the LL in the horizontal pipe are shown in Table 5.

## 3. Results and Discussion

### 3.1. Comparison of Simulation and Experimental Results

**Figure 7:**
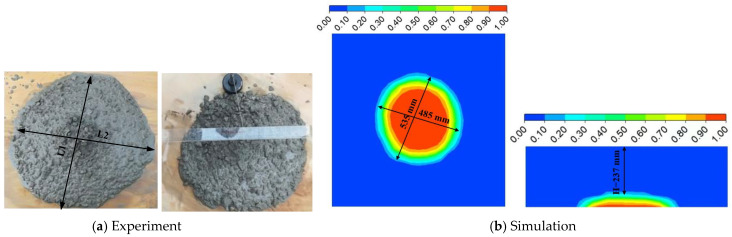
The slump test.

The experimental and numerical simulation results of the final flow form of fresh concrete in the slump test are shown in Figure 7. The average H and L_1_ × L_2_ of the experiment are 240 and 488 × 538 mm, respectively. The simulated H and L_1_ × L_2_ are 237 and 485 × 535 mm, respectively. As can be observed from the final flow form and the average H and L_1_ × L_2_, the simulation results were clearly close to the experimental results. Thus, the established model could well simulate the flow properties of fresh concrete in the slump test.

**Figure 8:**
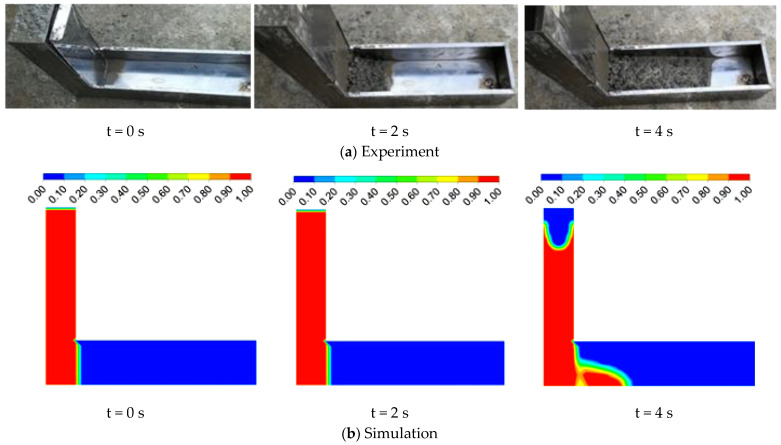
The flowing process of fresh concrete in the L-box test.

**Figure 9:**
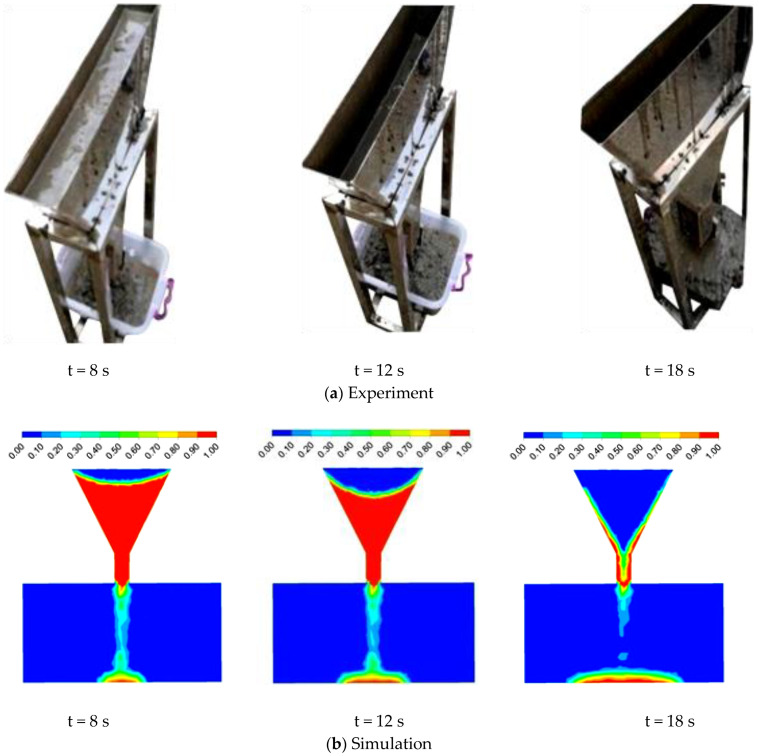
The flowing process of fresh concrete in the V-funnel test.

The experimental and simulation results of the L-box and V-funnel tests of fresh concrete are shown in Figure 8 and Figure 9, respectively. The simulation results for the flow of fresh concrete in both the L-box and V-funnel were consistent with the experimental results at the same flow times. The average B_m_ value from multiple experiments was 0.37, while the simulated B_m_ was 0.39. The variation range of T_v_ obtained from multiple experiments was between 16.5 and 18.8 s, with an average value of 17.9 s. The simulated T_v_ was 18 s.

The experiments and simulations of the slump, L-box flow, and V-funnel tests showed that the established CFD model using the Bingham model in commercial software ANSYS-Fluent v19 could well simulate the flow behavior and performance of fresh concrete.

### 3.2. Effect of T_LL_ on P_L_

**Figure 10:**
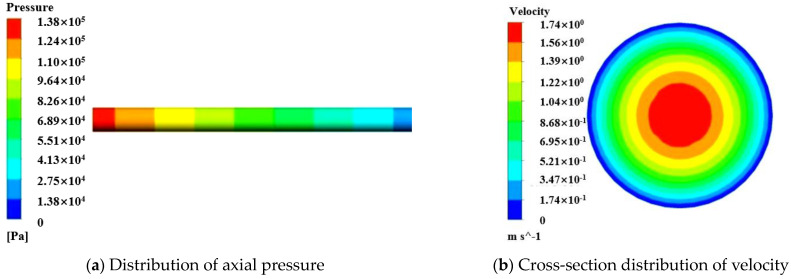
Simulation results of S50A20 pumping without LL.

**Figure 11:**
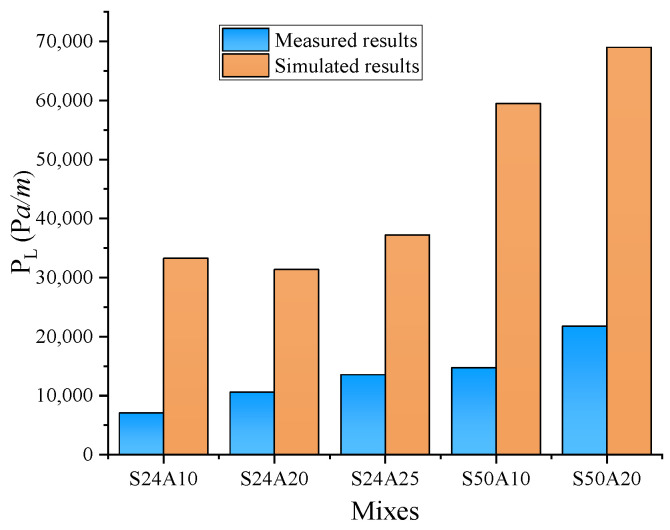
Comparison of simulation and measured P_L_ at flow rates of 40 m^3^/h without LL.

The flow of S50A20 in a horizontal pipe with an inlet flow rate of 40 m^3^/h was simulated. When the size of the T_LL_ was 0 mm, the yield stress and plastic viscosity of the fresh concrete were 80 Pa and 30 Pa.s, respectively. The simulated axial pressure contours and velocity in the pipe flow are shown in Figure 10. The axial pressure of the pipeline gradually decreased from the maximum pressure at inlet to 0 at outlet. The velocity of the concrete was highest at the center of the pipeline and lowest at the pipe wall. Moving from the center to the wall, the velocity of the concrete gradually decreased from the maximum to 0. Additionally, simulations were conducted for concrete pumping at a flow rate of 40 m^3^/h when the T_LL_ was 0 mm. The simulation results for the P_L_ were compared with the experimental P_L_ data from Choi et al. [23] and are shown in Figure 11. It was found that the simulated results differed significantly from the experimental results when the T_LL_ was 0 mm, being approximately three times larger. This means concrete cannot be pumped without the formation of an LL at the interface between the concrete and the pipe wall. Therefore, to ensure the pumpability of fresh concrete in actual construction, an LL with the appropriate thickness and stable state should be formed on the pipe wall during the pipeline flow to reduce the effect of friction. Similarly, the influence of LL on the pumpability of concrete should also be considered in the numerical simulation.

**Figure 12:**
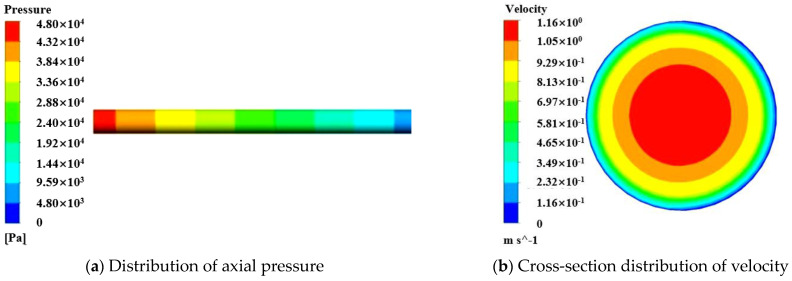
Simulation results of S50A20 pumping considering LL.

**Figure 13:**
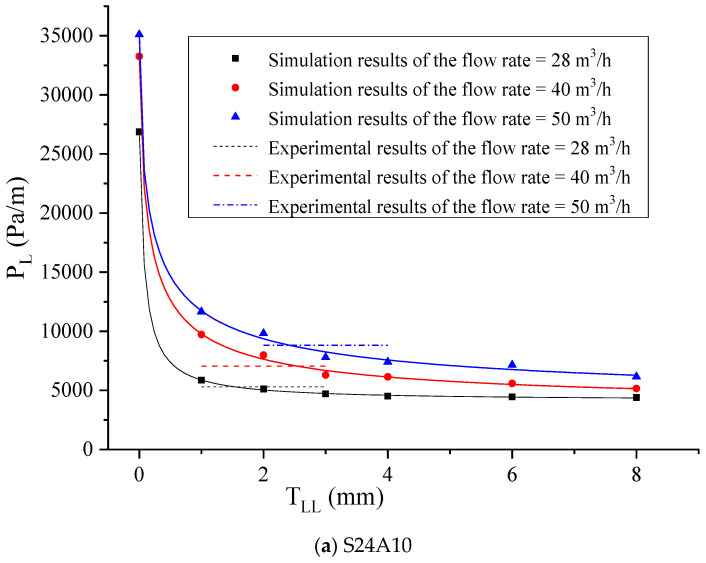

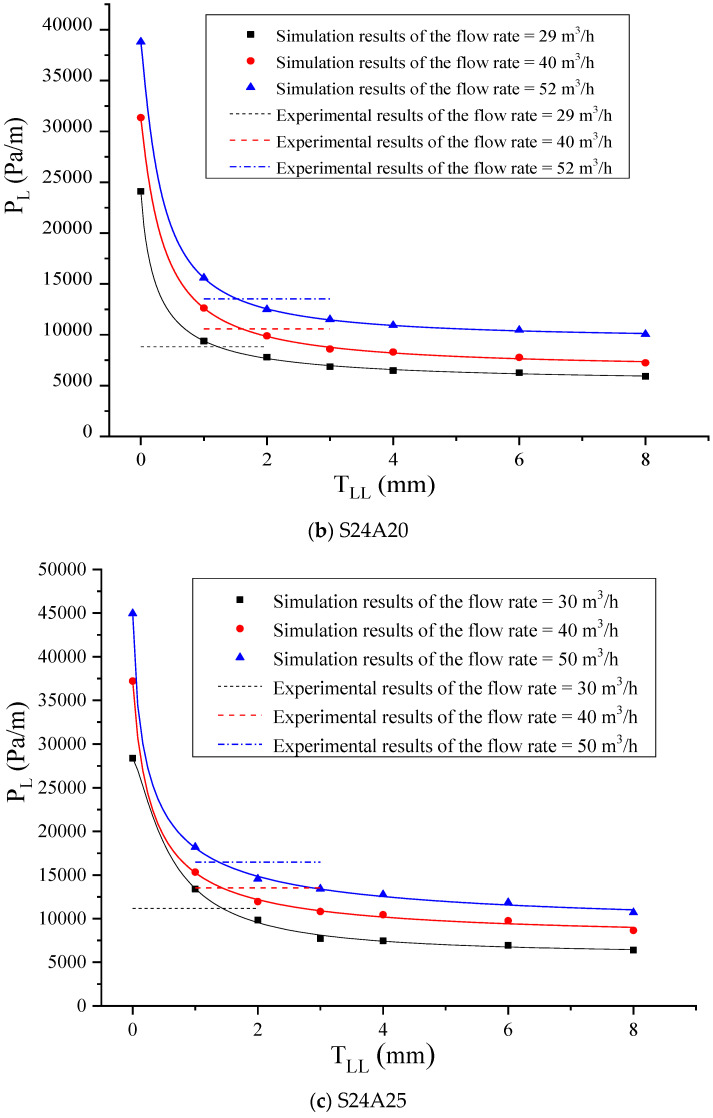

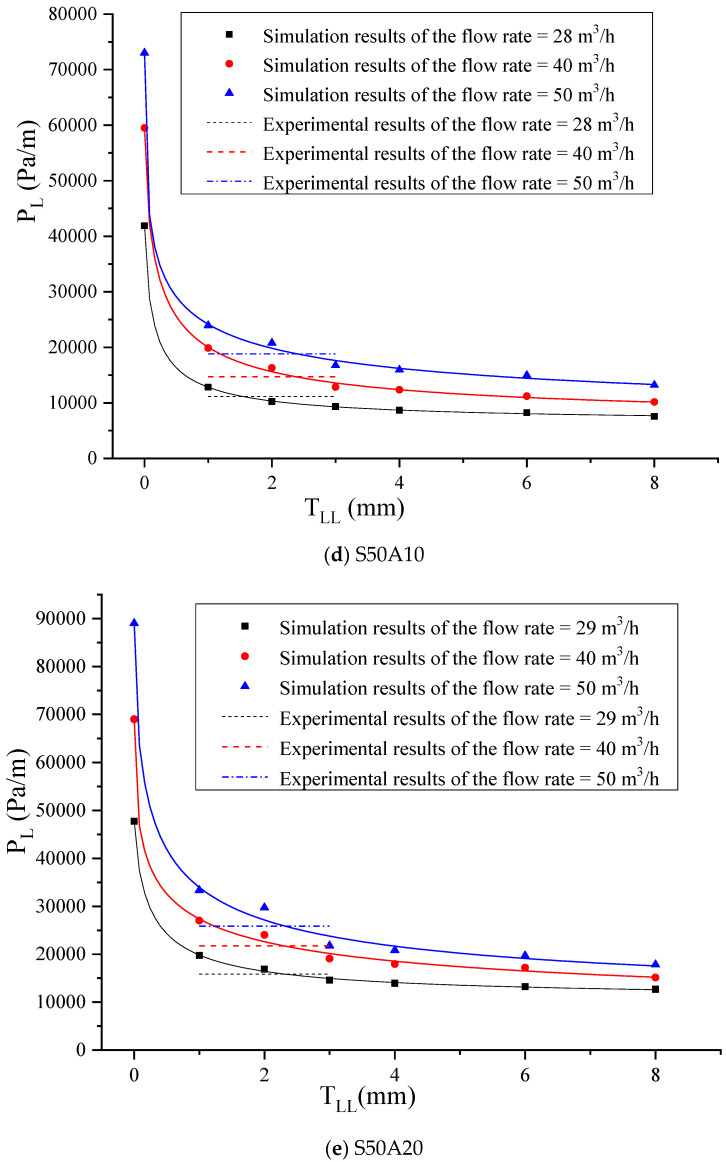

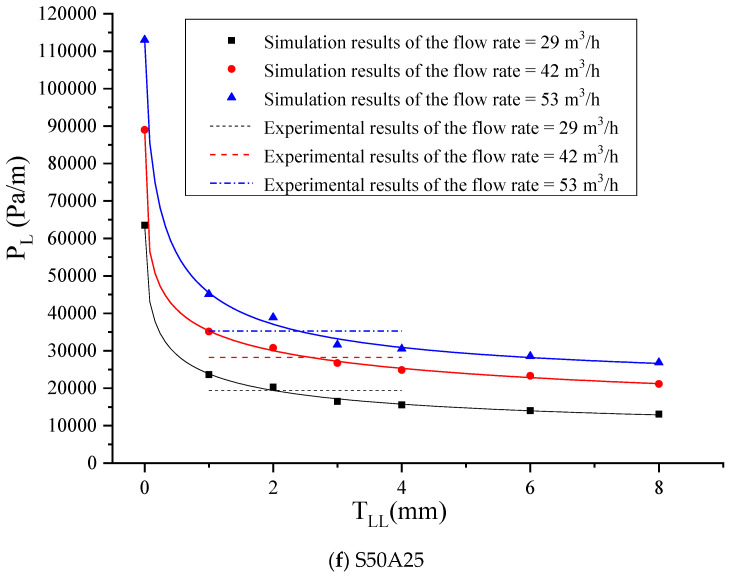
Comparison of simulation and measured P_L_.

The pumping of S50A20 in a horizontal pipe with an inlet flow rate of 40 m^3^/h and a T_LL_ of 2 mm was simulated. The yield stress and plastic viscosity of the central-layer concrete and the LL mortar were set at 80 Pa and 30 Pa.s and 12 Pa and 2 Pa.s, respectively. The contours of pressure and velocity in the pipe flow were obtained and are shown in Figure 12. Compared to Figure 10, it was found that the formation of the LL significantly reduces the pressure required for the flow of the fresh concrete in the pipe and the maximum speed of the central concrete. To ensure the pumpability of fresh concrete in actual construction, an LL should be formed on the pipe wall. Figure 13 presents a comparison between the simulated P_L_ for different T_LL_ values (0, 1, 2, 3, 4, 6, and 8 mm, respectively) and the measured P_L_ for six types of concrete with different rheological parameters under specific flow rates. The simulated results showed the P_L_ decreased with the increase in the T_LL_. However, the degree of reduction in the P_L_ gradually decreased with the increase in the T_LL_. When the T_LL_ was increased from 0 to 2 mm, the effect was more significant, reducing the P_L_. Especially, the formation of the LL on the pipe wall could largely reduce the P_L_, even if it is a thin layer. However, when the T_LL_ exceeded 4 mm, the influence of the continuous increase in the T_LL_ on the P_L_ was smaller. Combining the nonlinear fitted curve describing the simulated results and the horizontal line expressing the experimental results, the value of the abscissa of their intersection point was obtained, which represents the size of the T_LL_. For pumping the S24A10, when the flow rate was 28, 40, and 50 m^3^/h, the obtained value of the T_LL_ was 1.54, 2.53, and 2.41 mm, respectively. For pumping the S24A20, when the flow rate was 29, 40, and 52 m^3^/h, the obtained value of the T_LL_ was 1.23, 1.8, and 1.53 mm, respectively. For pumping the S24A25, when the flow rate was 30, 40, and 50 m^3^/h, the obtained value of the T_LL_ was 1.43, 1.42, and 1.38 mm, respectively. For pumping the S50A10, when the flow rate was 28, 40, and 50 m^3^/h, the obtained value of the T_LL_ was 1.53, 2.37, and 2.39 mm, respectively. For pumping the S50A20, when the flow rate was 29, 40, and 50 m^3^/h, the obtained value of the T_LL_ was 2.31, 2.3, and 2.31 mm, respectively. For pumping the S50A25, when the flow rate was 29, 42, and 53 m^3^/h, the obtained value of the T_LL_ was 2, 2.58, and 2.39 mm, respectively. The size of the T_LL_ was between 1 and 3 mm, and the minimum and the maximum were 1.23 and 2.58 mm, respectively. Their average value was 1.97 mm. The determined value of the T_LL_ is shown in Table 6.

**Figure 14:**
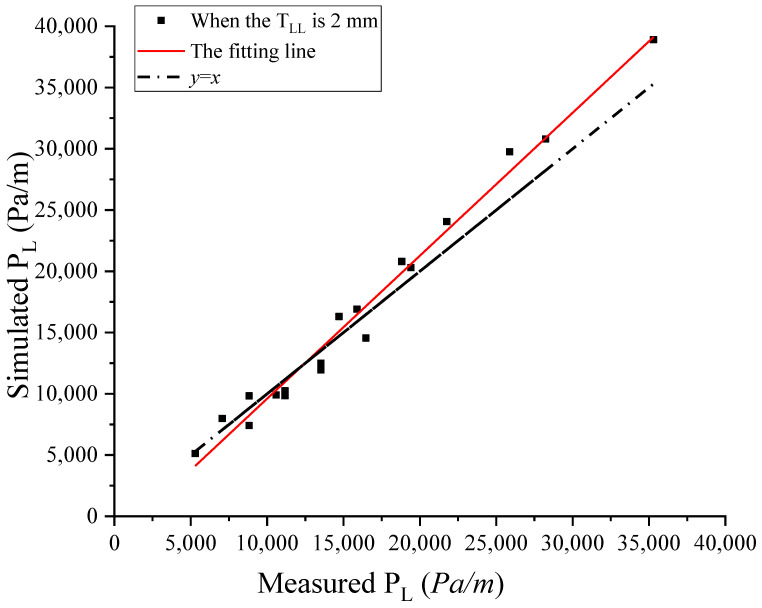
Comparison of simulation and measured pumping P_L_ when T_LL_ = 2 mm.

**Figure 15:**
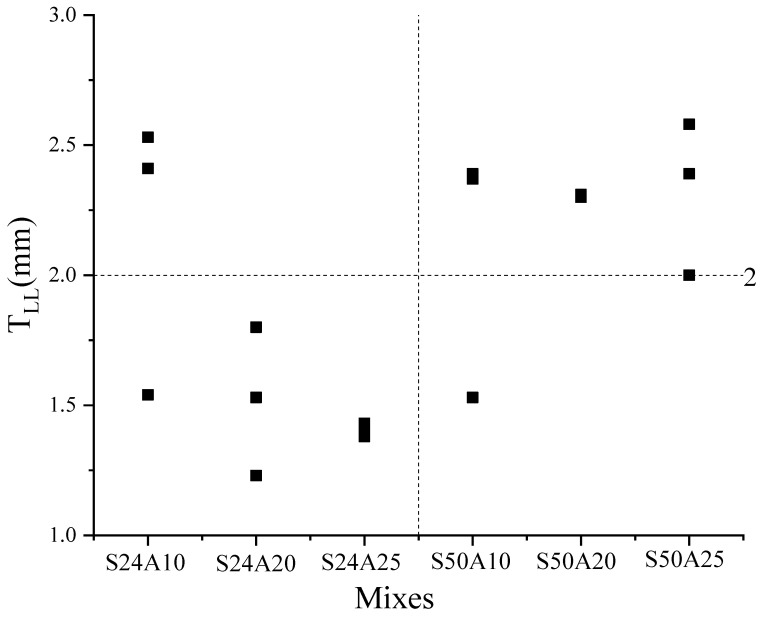
The effect of concrete types on the T_LL_.

**Figure 16:**
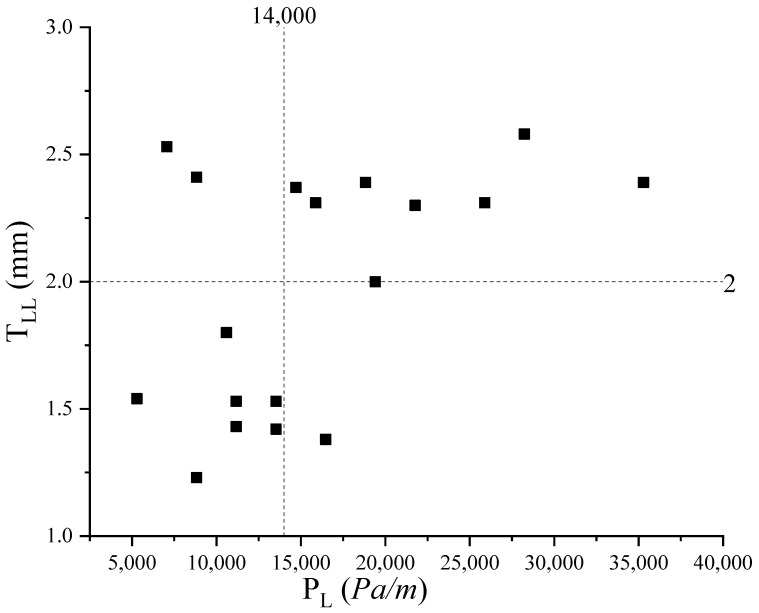
The relationship between the P_L_ and the T_LL_.

The fitting line between the simulated P_L_ and the measured P_L_ when the T_LL_ was 2 mm is shown in Figure 14. It could be found that the error between the fitting line and the line of *y* = *x* was smaller. This indicated the simulation results were well correlated with the experimental results. In reference [24], a T_LL_ of about 2 mm was obtained by means of the particle image velocimetry technique, which is consistent with our conclusions. The effect of concrete types on the T_LL_ is shown in Figure 15. Regardless of the size of the aggregate and the flow rate pumped, 1 mm < T_LL_ < 2 mm for S24 concrete and 2 mm < T_LL_ < 3 mm for S50 concrete. However, the value of the T_LL_ was related to the strength (S24, S50), the size of the aggregate (A10, A20, A25), and the flow rate of pumping. The relationship between the P_L_ and the T_LL_ is shown in Figure 16; this relationship is similar to the effect of concrete types on the T_LL_. It was found that when the P_L_ was larger than 14,000 Pa/m, 2 mm < T_LL_ < 3 mm; conversely, 1 mm < T_LL_ < 2 mm. Relating Figure 15 to Figure 16, it was concluded that both the type of fresh concrete and the flow rate of pumping significantly affected the P_L_. Then, the P_L_ impacted the T_LL_. However, the T_LL_ also impacted the P_L_. Finally, this made the T_LL_ change within a certain range. The above findings also guide us in using CFD to simulate the flow of all types of concrete in the actual pumping pipeline with a T_LL_ of 2 mm to obtain their pumping pressure and guide the actual construction.

## 4. Conclusions

In this paper, flow tests such as the slump test, L-box test, and V-funnel test on fresh C30 concrete were conducted experimentally and simulated numerically employing the CFD method. The flow of six kinds of fresh concrete under three groups with different pumping flow rates and different T_LL_ values in the horizontal pipe was simulated. The main conclusions are summarized as follows:

The feasibility of simulating the rheological behavior and properties of fresh concrete employing the CFD method and the Bingham model was demonstrated through experiments and simulations of fresh concrete flow tests, such as the slump test, L-box test, and V-funnel test.

When the LL on the pipe wall was not considered, the simulation result of the P_L_ was approximately three times higher than the experimental results. Conversely, the formation of the LL significantly reduces the P_L_. Therefore, to ensure the pumpability of fresh concrete in actual pumping construction, an LL must form on the pipe wall. The T_LL_ significantly affects the P_L_. As the T_LL_ increases, the P_L_ decreases. However, the effect of increasing the T_LL_ on reducing the P_L_ gradually decreases. When the T_LL_ increased from 0 to 3 mm, the reduction in P_L_ was more pronounced. Especially, the formation of the LL could largely reduce the P_L_, even if it is a thin layer.

Relating the intersection point of the nonlinear fitted curve describing the simulated P_L_ and the horizontal line expressing the experimental P_L_, the T_LL_ for different flow rates for the six kinds of fresh concrete could be obtained. The values of the T_LL_ ranged between 1 and 3 mm, with the minimum, maximum, and average values being 1.23 mm, 2.58 mm, and 1.97 mm, respectively. It was also found that the strength (S24, S50), aggregate size (A10, A20, A25), and pumping flow rate all affected the T_LL_. The mechanism of action was that the type of fresh concrete and the flow rate of pumping significantly affected the P_L_. Then, the P_L_ impacted the T_LL_. However, the T_LL_ also impacted the P_L_. Finally, this made the T_LL_ change within a certain range. When P_L_ > 14,000 Pa/m, 2 mm < T_LL_< 3 mm; conversely, 1 mm < T_LL_< 2 mm. Therefore, we can use CFD to simulate the flow of all types of concrete in the actual pumping pipeline with a T_LL_ of 2 mm to obtain their pumping pressure and guide the actual construction.

## Figures and Tables

**Table 2:** The experimental results of the slump test.

Name	H (mm)	L_1_ × L_2_ (mm)
Number		
1	245	510 × 600
2	230	460 × 510
3	240	490 × 530
4	235	480 × 500
5	245	500 × 550
Average	239	488 × 538

**Table 3:** The experimental results of the L-box test.

Name	H_2_ (mm)	H_1_ (mm)	B_m_
Number			
1	40	105	0.381
2	37	102	0.363
3	38	110	0.345
4	40	103	0.388
5	39	105	0.372
Average	38.8	105	0.37

**Table 4:** The experimental results of the V-funnel test.

Name	T_v_ (s)
Number	
1	18.5
2	17.8
3	18.8
4	16.5
5	17.9
Average	17.9

**Table 5:** Rheological parameters of fresh concrete and LL during pumping test [23].

Mixes	Aggregate Size	A10		A20		A25	
Strength	Item	LL	Concrete	LL	Concrete	LL	Concrete
S24	Plastic viscosity (Pa.s)	0.5	8.0	0.8	10.0	1.0	13.0
	Yield stress (Pa)	15.0	300.0	12.0	200.0	5.0	150.0
S50	Plastic viscosity (Pa.s)	1.3	25.0	2.0	30.0	2.5	40.0
	Yield stress (Pa)	11.0	100.0	12.0	80.0	50.0	80.0

**Table 6:** Determined results of T_LL_.

Mixes		Measured Results [23]		Determined Results T_LL_ (mm)
Design Strength	Aggregate Size	P_L_ (Pa/m)	Flow Rate (m^3^/h)	
S24	A10	5294	28	1.54
		7059	40	2.53
		8824	50	2.41
	A20	8824	29	1.23
		10,588	40	1.8
		13,529	52	1.53
	A25	11,176	30	1.43
		13,529	40	1.42
		16,471	50	1.38
S50	A10	11,176	28	1.53
		14,706	40	2.37
		18,824	50	2.39
	A20	15,882	29	2.31
		21,765	40	2.3
		25,882	52	2.31
	A25	19,412	29	2
		28,235	42	2.58
		35,294	53	2.39
Average value				1.97

## Data Availability

The original contributions presented in the study are included in the article, further inquiries can be directed to the corresponding author.

## References

[1] Feys D., De Schutter G., Khayat K.H., Verhoeven R. (2016). Changes in rheology of self-consolidating concrete induced by pumping. Mater. Struct..

[2] Sustandi A., Arianti S., Grand W. (2020). Study of factors affecting productivity of pouring concrete using portable concrete pump in construction project X. IOP Conf. Ser..

[3] Kwon S.H., Jang K.P., Kim J.H., Shah S.P. (2016). State of the Art on Prediction of Concrete Pumping. Int. J. Concr. Struct. Mater..

[4] Secrieru E., Cotardo D., Mechtcherine V., Lohaus L., Schröfl C., Begemann C. (2018). Changes in concrete properties during pumping and formation of lubricating material under pressure. Cem. Concr. Res..

[5] Feys D., Khayat K.H., Khatib R. (2016). How do concrete rheology, tribology, flow rate and pipe radius influence pumping pressure?. Cem. Concr. Compos..

[6] Feys D., De Schutter G., Fataei S., Martys N., Mechtcherine V. (2022). Pumping of concrete: Understanding a common placement method with lots of challenges. Cem. Concr. Res..

[7] Chajec A., Šavija B. (2024). The effect of using surface functionalized granite powder waste on fresh properties of 3D-printed cementitious composites. J. Build Eng..

[8] Chajec A. (2023). The use of granite powder waste in cementitious composites. JMRT.

[9] Kaplan D. (1999). Pumping of Concrete. Ph.D. Thesis.

[10] Choi M., Roussel N., Kim Y., Kim J. (2013). Lubrication layer properties during concrete pumping. Cem. Concr. Res..

[11] Yammine J., Chaouche M., Guerinet M., Moranville M., Roussel N. (2008). From ordinary rheology concrete to self-compacting concrete: A transition between frictional and hydrodynamic interactions. Cem. Concr. Res..

[12] Choi M.S., Park S.B., Kang S.T. (2015). Effect of the Mineral Admixtures on Pipe Flow of Pumped Concrete. J. Adv. Concr. Technol..

[13] Ngo T.T., Kadri E.H., Bennacer R., Cussigh F. (2009). Use of tribometer to estimate interface friction and concrete boundary layer composition during the fluid concrete pumping. Constr. Build. Mater..

[14] Ngo T., Kadri E., Cussigh F., Bennacer R., Duval R. (2010). Practical tribometer to estimate pumpability of fresh concrete. J. Asian Archit. Build..

[15] Feys D. (2009). Interactions between Rheological Properties and Pumping of Self-Compacting Concrete. Ph.D. Thesis.

[16] Feys D., Khayat K.H., Perez-Schell A., Khatib R. (2015). Prediction of pumping pressure by means of new tribometer for highly-workable concrete. Cem. Concr. Compos..

[17] Chen L., Liu G., Cheng W., Pan G. (2016). Pipe flow of pumping wet shotcrete based on lubrication layer. Springerplus.

[18] Kaplan D., de Larrard F., Sedran T. (2005). Design of concrete pumping circuit. ACI Mater. J..

[19] Koehler E.P., Fowler D.W., Ferraris C.F., Amziane S. (2006). A new, portable rheometer for fresh self-consolidating concrete. ACI Mater. J..

[20] Mechtcherine V., Nerella V.N., Kasten K. (2014). Testing pumpability of concrete using sliding pipe rheometer. Constr. Build. Mater..

[21] Feys D., Khayat K.H., Perez-Schell A., Khatib R. (2014). Development of a tribometer to characterize lubrication layer properties of self-consolidating concrete. Cem. Concr. Compos..

[22] Jo S.D., Park C.K., Jeong J.H., Lee S.H., Kwon S.H. (2012). A computational approach to estimating a lubricating layer in concrete pumping. Comput. Mater. Contin..

[23] Choi M.S., Kim Y.J., Kwon S.H. (2013). Prediction on pipe flow of pumped concrete based on shear-induced particle migration. Cem. Concr. Res..

[24] Le H.D., Kadri E.H., Aggoun S., Vierendeels J., Troch P., De Schutter G. (2015). Effect of lubrication layer on velocity profile of concrete in a pumping pipe. Mater. Struct..

[25] Met-flow S.A. (2002). Model UVP-Duo with Software Version 3 User’s Guide.

[26] Feys D., Verhoeven R., de Schutter G. (2007). Evaluation of time independent rheological models applicable to fresh self-compacting concrete. Appl. Rheol..

[27] Ngo T.T., Kadri E.H., Cussigh F., Bennacer R. (2012). Relationships between concrete composition and boundary layer composition to optimise concrete pumpability. Eur. J. Environ. Civ. Eng..

[28] Secrieru E., Fataei S., Schröfl C., Mechtcherine V. (2017). Study on concrete pumpability combining different laboratory tools and linkage to rheology. Constr. Build. Mater..

[29] Jang K.P., Kim W.J., Choi M.S., Kwon S.H. (2018). A new method to estimate rheological properties of lubricating layer for prediction of concrete pumping. Adv. Concr. Constr..

[30] Kwon S.H., Park C.K., Jeong J.H., Jo S.D., Lee S.H. (2013). Prediction of Concrete Pumping: Part II—Analytical Prediction and Experimental Verification. ACI J..

[31] Feys D., De Schutter G., Verhoeven R. (2012). Parameters influencing pressure during pumping of self-compacting concrete. Mater. Struct..

[32] Secrieru E., Khodor J., Schröfl C., Mechtcherine V. (2018). Formation of lubricating layer and flow type during pumping of cement-based materials. Constr. Build. Mater..

[33] Cui W., Zhao C., Wang S. (2021). Estimation of super high-rise pumping pressure for high-performance concrete based on computational fluid dynamics modeling and situation measurement. Adv. Civ. Eng..

[34] Feys D., Khayat K.H. (2015). Recent developments in evaluating pumping behavior of flowable and self-consolidating concrete. J. Sustain. Cem-Based. Mater..

[35] Sassi R., Jelidi A., Montassar S. (2023). Numerical simulation of fresh concrete flow in the L-box test using computational fluid dynamics. Mag. Concrete Res..

[36] Cao G., Bai Y., Shi Y., Li Z., Deng D., Jiang S., Xie S., Wang H. (2024). Investigation of vibration on rheological behavior of fresh concrete using CFD-DEM coupling method. Constr. Build. Mater..

[37] Ansys Inc. (2023). Ansys Fluent Theory Guide.

